# Cecal volvulus following mini gastric bypass: A case report and review of literature

**DOI:** 10.1016/j.ijscr.2018.11.024

**Published:** 2018-11-24

**Authors:** Hanan M. Alghamdi, Shadi AlShammary, Haitham Lardhi, Wafa AlDhafeeri, Noor AlLababidi

**Affiliations:** King Fahad Hospital of the University, Department of Surgery, College of Medicine, University of Imam Abdurahman Bin Faisal, Saudi Arabia

**Keywords:** Cecal volvulus, Mini gastric bypass, Bariatric surgery, Post op complication, Intestinal obstruction

## Abstract

Introduction: With the rising worldwide obesity epidemic, bariatric surgeries are gaining popularity as the most effective modality for achieving long term results in weight loss and reducing its metabolic sequels.

Case presentation: A 36-year-old female presented with a sudden onset of severe abdominal pain for 6 h. The patient underwent Mini gastric bypass (MGB) 2 years prior to presentation. Computed tomography (CT) scan revealed a 14-cm dilatation of the cecum occupying the left upper quadrant of the abdomen with the swirling appearance of the mesentery. These findings were confirmed through emergency exploratory laparotomy to be cecal volvulus. a right hemicolectomy and conversion of MGB to a conventional Roux-en-Y gastric bypass.

Discussion: a modification of the classical gastric bypass surgery the MGB considered by its advocates to be a safer, simpler, and more effective option. However, serious complications can take place especially in the early learning curve of the procedure.

**Conclusions:**

We present a unexpected acute in top of chronic cecal volvulus in patient two years post MGB. The uncommon serious complication should be considered one of the differential diagnosis of acute surgical abdomen in those patients.

## Highlights

• Cecal volvulus is one of the differential diagnosis of post Mini-gastric bypass acute intestinal obstruction. and pain.
• Urgent intervention in acute presentation post gastric bypass is the key to saving the patient life and lower morbidity.
• Conversion of Mini-gastric bypass to Roux-en-Y gastric bypass in any complication surgery is recommended when feasible.

## 1 Introduction

With the rising worldwide obesity epidemic, bariatric surgeries are gaining popularity as an effective modality for achieving long term results [1]. Single Anastomosis Gastric Bypass (Omega Loop Gastric Bypass/Mini-gastric Bypass /Single-Anastomosis Bypass) considered a modification of the classical gastric bypass surgery. A long, vertical gastric tube is made along the lesser curvature of the stomach, with a gastroenterostomy 180–200 cm distal to the ligament of Treitz [2]. Compared to various approaches, MGB is considered a safer, simpler, and more effective option [3,4]. Nonetheless, the reported complications of MGB include severe gastritis, marginal ulcers, malnutrition, anastomosis leak, and small bowel obstructions [5]. Cecal volvulus has been reported by many case reports and case series as a considerable cause of abdominal pain in patients who underwent Roux-en-Y gastric bypass (RYGB) [6,7] or gastric banding procedure [8]. This case is the of cecal volvulus post-MGB aimed to raise knowledge and precaution.

The presented case details a patient with cecal volvulus and an internal hernia post MGB procedure who presented with acute abdominal pain to the emergency department of King Fahad Hospital of the University in Khobar, Saudi Arabia. Written consent was obtained from the patient for publication of this case report. Our case has been reported in line with the SCARE criteria [9].

## 2 Case presentation

A 36-year-old female presented to the emergency department two months post caesarean section with a sudden onset of severe abdominal pain for 6 h. The pain was associated with two episodes of vomiting, abdominal distension and constipation. The patient underwent MGB 2 years prior to presentation. In addition, she was complaining of severe dyspepsia and unsatisfactory weight loss following her MGB. She has no known chronic medical illnesses. She had undergone open cholecystectomy 12 years ago.

On physical examination, she was afebrile, tachycardic with normal blood pressure. Abdominal examination showed right-sided abdominal fullness and tenderness and empty rectum on digital rectal examination.

**Fig. 1:**
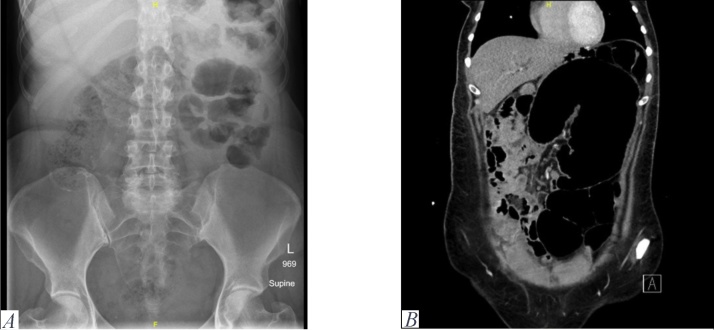
Radiological studies. A) Abdominal X-ray image showing dilated intestinal loops on the left side. B) Abdominal computed tomography image showing a 14-cm dilatation of the cecum occupying the left upper quadrant of the abdomen.

**Fig. 2:**
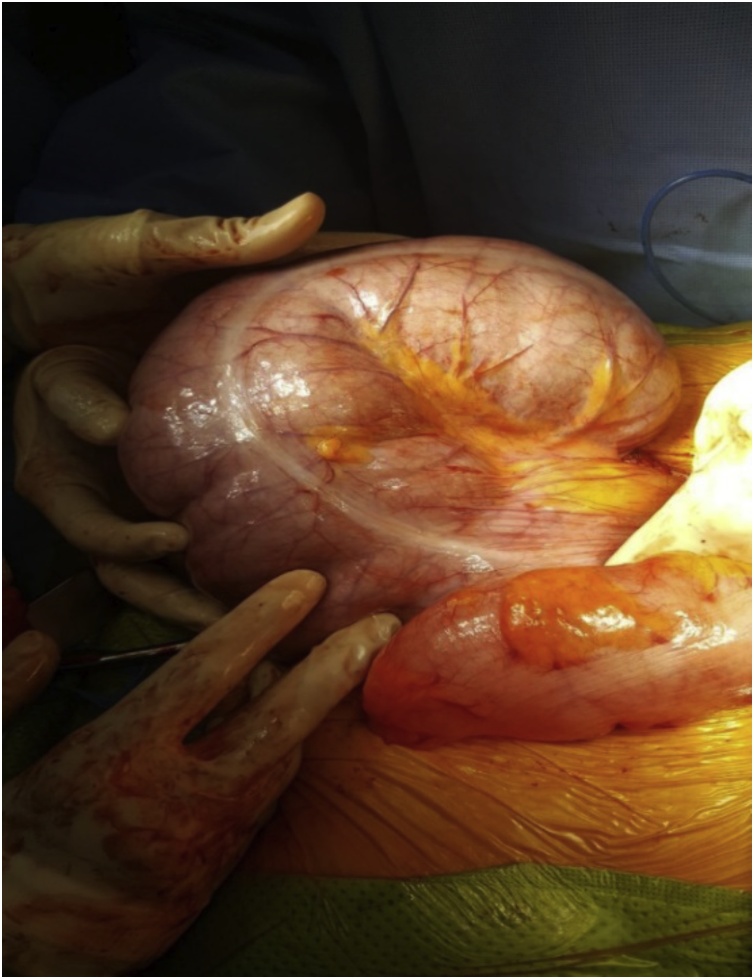
Intraoperative picture showing Cecal dilatation and volvulus.

Radiological studies including abdominal X-ray (Fig. 1) and computed tomography (CT) scan (Fig. 1) revealed a 14-cm dilatation of the cecum occupying the left upper quadrant of the abdomen with the swirling appearance of the mesentery. These findings were confirmed through emergency exploratory laparotomy. Intraoperatively, the cecum was still viable but severely dilated and twisted (Fig. 2). An incarcerated Petersen’s hernia was found with no signs of strangulation. At laparotomy, she underwent a right hemicolectomy, reduction of Petersen’s hernia, and conversion of MGB to a conventional retrocolic Roux-en-Y gastric bypass with closure of the mesentric defect. The post-operative period went uneventful and she was discharged from the hospital in good condition. Post-operative follow-up at 24 months showcased satisfactory weight loss and improvement of dyspepsia symptoms with no recurrence of bowel obstruction symptoms.

## 3 Discussion

With bariatric surgeries being widely performed, the incidence of complications related to these procedures continues to rise. Bowel obstruction is a life threatening complication that may occur with bariatric surgeries [5]. The differential diagnoses for bowel obstruction following bariatric procedures includes obstruction secondary to technically narrow bypass or anastomosis, internal bowel herniation, port site hernia, and adhesive small bowel obstruction. We would like to emphasize once more that cecal volvulus has been reported by several case reports and in case series as a serious cause of abdominal pain in patients who underwent RYGB [6,7] or gastric banding procedure [8].

Although internal hernias are very rare, Petersen’s hernia (PH) accounts for more than 50% of the cases [10]. PH occurs by migration of an intestinal loop into a potential space posterior to a gastrojejunostomy, creating an internal hernia [11]. The presumptive absence of internal hernias in the post-operative follow-up period is one of the advantages of MGB over RYGB, according to MGB advocates [[12], [13], [14]]. Yet this case, as well as two other recent case reports, indicate that MGB could be complicated by internal hernias [15,16]. However, the data is not sufficient to recommend closure of the mesenteric defect intraoperatively.

Abdominal pain was the main clinical presentation in this case of cecal volvulus, which can be either chronic constant or intermittent upper quadrant pain as reported in a case series [7]. In comparison, midgut volvulus presents with nonspecific or central abdominal pain [17,18]. PH presents with sudden diffuse or epigastric pain, which is usually described as sharp and cramping and can be associated with nausea [15,19]. These complications can be life-threating. All may present with non-specific abdominal pain, making them important to include them in the list of differential diagnoses in abdominal pain post-MGB. The timing at which complications may appear is also important, as cecal volvulus usually presents between 2–3 years after bariatric surgery [7].

Upon reviewing reported gastric bypass cases, CT was found to be the preferred modality to investigate abdominal pain and suspected bowel obstruction, and radiological findings were sufficient to confirm the presence of midgut malrotation [17,18]. Surprisingly, in one case, CT was not able to detect cecal volvulus pre-operatively in the reported cases [7]. This raises a question regarding the sensitivity of CT in nonspecific abdominal pain in MGB patients to detect cecal volvulus. PH however, was accurately diagnosed by examining CT findings [15]. Furthermore, CT was superior to other radiological modalities, including abdominal X-ray, ultrasound, and upper endoscopy, all of which were unremarkable in comparison [15,19].

Cecal volvulus could present in a considerably critical stage, when emergency surgical intervention is needed. When proceeding to exploratory laparotomy, the type of surgical management is dependent on the degree of ischemia and bowel gangrene. Resection and anastomosis, the surgical treatment of gangrenous bowel, is also equally recommended in viable bowel since it is associated with less recurrence rate [20]. Cecopexy and cecostomy can be used when the wall is viable and with normal thickness [21].

In some cases, laparoscopic cecopexy has been sufficient for the treatment of non-gangrenous cecal volvulus post RYGB [7]. If Petersen’s hernia is present, reduction and repair of the defect is recommended to prevent recurrence [15]. In a reported case, a side-to-side enteroenterostomy was performed to reconnect the GB [19]. MGB carried a greater risk for complications such as bile reflux gastritis, severe chronic diarrhea, and severe protein malnutrition compared to other bariatric surgeries. In two recent case reports, the conversion of MGB to RYGB was done due to either failure of weight loss or severe complications, such as the one reported in our case [21,22].

In conclusion, differential diagnoses of bowel obstruction after bariatric surgery should remain wide with the rising number of cases performed. Cecal volvulus should be considered as a potential cause of abdominal pain post-MGB that warrants urgent diagnosis and management to avail fatal sequels.

## Conflicts of interest

None.

## Funding

This research did not receive any specific grant from funding agencies in the public, commercial, or not-for-profit sectors.

## Ethical approval

Case reports are exempted from ethical approval according to policies of Imam Abdulrahman Bin Faisal University.

## Consent

Written informed consent was obtained from the patient for publication of this case report.

## Author contribution

**Hanan AlGhamdi:* design the study, data collection, writing the manuscript, performed the operation, reviewing the final manuscript of the case report, final approval.*

*Wafa Aldhafeeri*: study design, data collection, reviewing.

*Noor Allababidi:* study design, data collection, writing the paper.

*Shadi Alshammary:* study concept, reviewing article, correction and editing of the case report.

*Haitham Lardhi:* study concept, reviewing article, correction and editing of the case report.

## Registration of research studies

N/A.

## Guarantor

Dr. Hanan Alghamdi.

## Provenance and peer review

Not commissioned externally peer reviewed.
